# The Prevalence of Polycystic Ovary Syndrome in Women with Idiopathic Intracranial Hypertension

**DOI:** 10.6064/2012/708042

**Published:** 2012-07-11

**Authors:** Inbal Avisar, Dan D. Gaton, Hirsch Dania, Hadas Stiebel-Kalish

**Affiliations:** ^1^Department of Ophthalmology, Rabin Medical Center, Beilinson Campus, 49100 Petah Tiqva, Israel; ^2^Sackler Faculty of Medicine, Tel Aviv University, 69918 Tel Aviv, Israel; ^3^Department of Endocrinology, Rabin Medical Center, Beilinson Campus, 49100 Petah Tiqva, Israel

## Abstract

*Purpose*. The purpose of this study is to re-evaluate whether the prevalence of polycystic ovary syndrome (PCOS) amongst women with idiopathic intracranial hypertension (IIH) is higher than in the general population using the NIH criteria. *Methods*. We included all consecutive women with IIH of child-bearing age seen at a hospital-based neuro-ophthalmology clinic between the years 2000–2005. All consenting women included in this study filled-out a screening questionnaire aimed at identifying women at risk for PCOS. The endocrinologist examined each patient suspect of PCOS as well as their biochemical results and diagnosed PCOS according to NIH criteria. The prevalence of PCOS in these women with IIH was compared with the reported prevalence of PCOS in the general population. *Results*. Out of 58 women with IIH who completed the study, definite PCOS was diagnosed in nine women (9/58; 15.5%). We found a significantly higher prevalence of PCOS using the NIH criteria among the IHH study group (15.5%, *P* = 0.001) compared to the general population (8.7%). *Conclusions*. The prevalence of PCOS is higher among patients with IHH, compared to the general population. We suggest a novel screening questionnaire to aid in the identification of women with IIH at risk for PCOS.

## 1. Introduction

The etiology of idiopathic intracranial hypertension (IIH), also known as pseudotumor cerebri, is unclear. IIH occurs mainly in young obese women. Underlying and associated endocrinological abnormalities have been reported in numerous studies [1, 2].

Diagnostic criteria for IIH include signs and symptoms that reflect intracranial pressure (e.g., papilledema), documented elevated intracranial pressure with normal CSF composition, no evidence of mass, structural, or vascular lesion on MRI, MR venography, or contrast-enhanced CT, and no other cause of intracranial hypertension [1, 3]. Treatment is aimed mainly to prevent visual compromise and alleviate headaches. Acetazolamide, weight loss, and correction of underlying risk factors, including endocrinological abnormalities, are recommended. Some cases are refractory to medical intervention and require surgery (fenestration of the optic-nerve sheath or lumboperitoneal shunting) [4, 5]. Women with IIH need all the help we can offer to aid them in fighting obesity and endocrinological abnormalities in order to prevent visual compromise.

The polycystic ovary syndrome (PCOS) includes truncal obesity, menstrual irregularities, hyperandrogenism with hirsutism, acne, alopecia, and multiple ovarian follicular cysts. PCOS is characterized by several endocrine abnormalities, including increased conversion of androstenedione to estrone by stromal cells within adipose tissue [6, 7]. Insulin resistance is more common in PCOS [8, 9].

There are two main accepted systems for defining PCOS. According to the NIH criteria, two conditions have to be met: chronic anovulation and clinical or biochemical evidence of hyperandrogenism. Recently, the European Society for Human Reproduction and Embryology and the American Society for Reproductive Medicine (ESHRE/ASRM) suggested new criteria for the definition of PCOS. This is now defined as the presence of any two of the following conditions: (i) polycystic ovaries; (ii) oligo-/anovulation; (iii) clinical or biochemical evidence of hyperandrogenism [10]. There is still debate regarding the most appropriate criteria [11].

 Glueck et al. [12, 13] reported an increased prevalence of PCOS in women with IIH, compared to the prevalence of PCOS in the general unselected population [14].

Cosar et al. [15] found supporting evidence, using MRI and MR venography studies in women with PCOS and headache, that PCOS may facilitate the development of IHH.

We also suggest a screening questionnaire for the use of neurologists and ophthalmologists to aid in identifying women with IIH at risk for PCOS. Identifying women suspect of PCOS may aid those in need of further endocrinological workup and tailor treatment in this specific subgroup.

## 2. Methods

### 2.1. Participants

We included all consecutive women of child-bearing age who met the diagnostic criteria for IIH followed at our hospital-based neuroophthalmology clinic between the years 2000–2005.

### 2.2. Study Protocol

All consenting women included in this study filledout a screening questionnaire (Appendix), aimed at identifying women at risk for PCOS. Women with suspected PCOS, based on the questionnaires' results, underwent testing for LH, FSH, testosterone, and 17-hydroxy-progesterone serum levels. The endocrinologist examined each patient suspect of PCOS, as well as the biochemical results, and classified women as: (a) definite PCOS according to the NIH criteria if both conditions (anovulation and hyperandrogenism) were met. These women were referred for US to confirm polycystic ovaries and to serve as a base line for future followup. (b) Not meeting criteria for PCOS (only one criteria was met: oligo-/anovulation or clinical/biochemical hyperandrogenism), these patients were not referred to US.

The prevalence of PCOS in these women with IIH was compared with the reported prevalence of PCOS in the general population. We compared our results with a recent study, which is practically the largest community-based prevalence study of PCOS in a Caucasian population, presented by March et al. [16] The parameters of age, gender, height, and weight were compared with both groups.

The study design was approved by the institutional review board. Patients were asked to provide their consent to the filling of the questionnaire and to participate in study.

### 2.3. Statistical Analysis

A comparison of the prevalence of PCOS in these women with IIH with the reported prevalence of PCOS in the general population [16] was performed using *χ* ^2^  test. A *P* value of <0.05 was considered significant.

## 3. Results

We included in the study consecutive Caucasian women, aged 18–50 years (mean = 34.49, SD = 10.37), who met the diagnostic criteria for IIH and were followed and treated at the neuroophthalmologic clinic, Rabin Medical Center, Israel, between 2000–2005. Patient characteristics are listed in Table 1.

During 2000–2005, 136 consecutive patients met the diagnostic criteria for IIH. Sixty four (47%) of them were women, two of whom deceased during the study and four refused to fill out our questionnaire. Fifty-eight women were included in our study. Follow up ranged between 2 and 11 years (mean = 5; SD = 1.86).

Endocrinological abnormalities reflecting PCOS are listed in Table 2. The questionnaire revealed 22 of the 58 women (38%) as being at risk for PCOS (all had either oligo-/anovulation or clinical signs of hyperandrogenism). After reviewing the results of the questionnaires, blood tests and physical examination results, nine (9/58; 15.5%) had definite PCOS. US proved the presence of polycystic ovaries in all these women. 13 women (13/58; 8.7%) met only one criteria for PCOS (only oligo-/anovulation with no clinical or biochemical signs of hyperandrogenism). They were not referred to US, however we could rule out PCOS according to the NIH criteria. Overall PCOS was ruled out in 49 (84%) women according to the NIH criteria.

When comparing the results of the current study to previous studies in the general population [16], we found no difference in age, weight, and BMI parameters (*P* < 0.001 in all parameters). When comparing the values found in our study to this previous report, having the same age, weight and BMI distribution, we found a significantly higher prevalence of PCOS using the NIH criteria among the IHH study group (15.5%) compared to the general population (8.7%) [16].

The positive predictive value of the proposed screening tool aimed to asses PCOS risk using the questionnaire, based on the comparison between the 22 women suspected as harboring PCOS, and the 9 found to have PCOS based on NIH criteria were 41% (9/22).

Ten (17.2%) patients were found to have various other endocrinological abnormalities: four (7%) with hypothyroidism and six (10%) with diabetes (three of them with definite PCOS). Hyperlipidemia was found in seven patients (12%).

## 4. Discussion

We examined the use of a new screening questionnaire to identify women with IIH at risk for PCOS for the use of neuroophthalmologists. This questionnaire found that 38% of women with IIH were at risk to have PCOS. Further blood tests and an endocrinologist examination helped to define the diagnosis in 15.5% of patients included in the study. The positive predictive value of the proposed screening tool aimed to asses PCOS risk using the questionnaire was 41% (9/22). In addition, we found a significantly higher prevalence of PCOS using the NIH criteria among the IHH study group (15.5%) compared to the general population.

The NIH diagnostic criteria for the diagnosis of PCOS were based on a consensus of experts, who concluded that women have PCOS if they present with the combination of chronic oligo- or anovulation and clinical or biochemical signs of hyperandrogenism. We based our questionnaire on their concept which maintains that androgen excess is a central feature of the disease.

Ultrasound and blood tests were not carried out on women who did not present with clinical symptoms of PCOS, as was also the case in other community studies of PCOS prevalence [14, 16]. There may have been some women in this group who had hyperandrogenemia and polycystic ovaries but literature suggests that this is likely to be less than 1% of those with PCOS [14, 16].

When comparing the results of the current study to previous studies in the general population having the same age and BMI distribution, we found a higher prevalence of PCOS using the NIH criteria among the IHH study group (15.5%) compared to the general population (8.7%) [16]. In three other previous studies using the NIH criteria, values were even lower standing at 6.5–6.8% [17–19].

The results of this study confirm a previously reported association between IIH and PCOS. Glueck et al. [12] reported that in 38 women with IIH, 15 (39%) were found to have PCOS. In another study of 65 women with IIH, 37 (57%) were found to have PCOS [13]. Knochenhauer et al. [14] stated that this prevalence of PCOS (39%–57%) in women with IIH is 5 to 8 times greater than the 7% prevalence of PCOS in the general unselected population.

Cosar et al. [15] performed MRI and MR venography studies of the brain and orbit in women with PCOS and headaches and found that PCOS and headache are promoters of IHH.

Similar to Glueck's results, we found an association between PCOS and IIH, but in our group the prevalence was only nearly twice as common (15.5%) than the 8.7% prevalence of PCOS in the general unselected population [16].

On the basis of these findings, this study verifies the argument of Glueck et al. The difference in prevalence may be a result of Glueck's use of the Rotterdam criteria for PCOS which are not as strict as the NIH criteria.

We also found that 10 (17.2%) patients had other endocrinological abnormalities. This has been documented before [20, 21].

Our study group is relatively small in a bothersome way, but the group was compared to one of the most representative studies of PCOS prevalence in Caucasian women to date and was found comparative in terms of clinical parameters.

We suggest, for the first time, the use of a practical questionnaire that can be filled out by the patients themselves, as an aid in detecting PCOS risk in a neurology or ophthalmology clinic setting. In fact, this questionnaire is not of paramount importance by itself, since the patients can also be directly asked in case the neuroophthalmologist remembers the potential association between PCO and IHH. We did not validate formally this questionnaire as well. However, we offer this tool in order to emphasize the necessity in diagnosing the associating diseases in order to improve the management of IHH patients. Addressing PCOS may help with the treatment of IIH by aiding weight loss and managing associated endocrinological abnormalities such as glucose intolerance and hyperandrogenism. We call for further study of the benefit of PCOS treatment in the management of IIH.

## Figures and Tables

**Table 1:** Age, height, weight, and BMI of patients.

	Range	Mean	SD
Age (years)	18–50	34.49	10.37
Height (meters)	1.5–1.76	1.633	0.062
Weight (kg)	47–160	84.28	23.16
BMI (kg/m²)	18.3–58.7	26.49	19.37

**Table 2:** Endocrinological abnormalities reflecting PCOS.

Endocrinological abnormalities	No. (%)
Irregular period	13 (22.4%)
Fertility problems	9 (15.5%)
Hirsutism of face or body	17 (29.3%)
Acne	18 (31%)
Stria	13 (22.4%)
Hair loss/alopecia	25 (43%)

## Appendix

### A. The Questionnaire Used at this Study to Find Outpatients at Risk for PCOS

#### A.1. Part 1: Clinical Questionnaire

1. Height_________m
2. Weight_________kg
3. BMI_____________kg/m^2^ (calculated)
4. Age of first period_____________
5. Regularity of the menstrual cycle__________
6. Fertility problems_____________
7. Other systemic disorders (e.g.: diabetes, hyperthyroidism) __________

#### A.2. Part 2: Physical Signs

1. Hirsutism of face____________
2. Hirsutism of body___________
3. Acne______________________
4. Stria _____________
5. Hair loss_____________
